# Impact of Gestational Weight Gain on Delivery Outcomes in Women Undergoing Trial of Labour After Caesarean Section: A Cross-Sectional Study 

**DOI:** 10.7759/cureus.104918

**Published:** 2026-03-09

**Authors:** Rishita Modali, Bhabani Pegu, Indranil Das, Jenisha T Anitha, Anish Keepanasseril

**Affiliations:** 1 Obstetrics and Gynecology, Jawaharlal Institute of Postgraduate Medical Education and Research, Puducherry, IND

**Keywords:** body mass index, gestational weight gain, maternal and neonatal complications, previous cesarean section, tolac outcome

## Abstract

Background: Repeat cesarean deliveries are the primary driver of the global increase in caesarean section (CS) rates. Promoting trial of labour after caesarean (TOLAC) offers an opportunity to reduce unnecessary CS rates. While gestational weight gain (GWG) is a modifiable risk factor influencing maternal outcomes, its impact on TOLAC remains unclear.

Objective: To assess the association between abnormal GWG and the risk of failed trial of labour after CS.

Methods: A prospective case-control study was conducted at a tertiary care centre in India from January 2021 to December 2022. Women with a prior single CS and term singleton pregnancy undergoing TOLAC were included. Those with failed TOLAC were included as cases (*n* = 200), and those who had a successful vaginal birth after caesarean (VBAC) (*n* = 200) were included as controls. GWG was categorised per the Institute of Medicine (IOM) guidelines. Multivariate logistic regression analysis assessed the association between GWG and TOLAC outcomes, adjusting for key confounders.

Results: Excessive GWG was not significantly associated with TOLAC failure after adjustment for confounders (adjusted odds ratio (aOR), 95% confidence interval (CI)). A higher pre-pregnancy body mass index (BMI) (aOR, 95% CI) and the need for labour augmentation (aOR, 95% CI) increased the odds of TOLAC failure, whereas a prior successful VBAC reduced the risk. Neonatal outcomes did not differ significantly between groups.

Conclusions: In this cohort, excessive GWG did not increase the risk of failure after TOLAC. These findings suggest the EGWG should not deter anyone from offering a TOLAC, even those with higher pre-pregnancy BMI.

## Introduction

Globally, caesarean section (CS) remains one of the most common obstetric procedures. Although CS plays a critical role in improving maternal and neonatal outcomes, it carries the risk of maternal morbidity, with its impact persisting even on subsequent pregnancies, such as a morbidly adherent placenta [1]. In recent decades, there has been an increasing trend in the CS, with repeat caesarean being one of the major contributors to rising rates.  Promoting trial of labour after caesarean section (TOLAC) is an important strategy in reducing these rates [2,3].

Gestational weight gain (GWG) is linked to maternal and neonatal health and is guided by the Institute of Medicine’s (IOM) recommendations based on pre-pregnancy body mass index (BMI) [4]. Excessive GWG is associated with adverse outcomes like gestational diabetes, hypertensive disorders, and macrosomia, which can increase the likelihood of CS delivery [5]. The success of TOLAC depends on several maternal and obstetric factors, with GWG emerging as a modifiable risk factor [6]. Some studies suggested excessive GWG  increases the chances of a failure of TOLAC; others find no significant association [7]. This study evaluated whether abnormal GWG increases the chances of failure among women attempting TOLAC. 

## Materials and methods

Study design and setting  

This prospective, non-matched, case-control study was conducted at a tertiary care institute from January 2021 to December 2022. This regional perinatal teaching hospital manages high-risk pregnancies in the southeastern coastal region of India. It is also a referral centre in the region for pregnant women with previous caesarean section, where they are offered a TOLAC.  

Study procedure 

All consecutive pregnant women who underwent TOLAC, aged 18 years or older with a previous one caesarean delivery, at or more than 37 weeks of gestational age, were identified from the confinement register. Based on the outcome of the TOLAC, women who had a vaginal delivery, either spontaneous or assisted, were classified as cases, and controls were those who required an emergency caesarean section or underwent laparotomy for uterine rupture or other complications during the intrapartum or postpartum period. Those with incomplete documentation, including missing pre-pregnancy or first-trimester weight data, were excluded. The IOM for human studies approved the study (approval number: JIP/IEC/2020/0312).   

Exposure

Excessive GWG is considered the primary exposure of interest, calculated based on the BMI category, following the IOM recommendation for the BMI. GWG was calculated as the difference between weight at admission to delivery and the pre-pregnancy weight or in the first trimester (for those whose pregnancy data is unavailable; at 14 weeks or less period of gestation, when the GWG is negligible). 

Selection of cases and controls

During the study period, 24,901 deliveries were recorded, of which 3,063 were among women with a previous caesarean section. Repeat caesarean sections were performed without a TOLAC in 1724 (56.3%) cases. After excluding these cases, 1,339 (43.7%) underwent TOLAC, which included 138 preterm cases. Of the remaining 1,181 cases, 564 (47.8%) achieved a successful vaginal birth after caesarean (VBAC), while 617 (52.3%) required an emergency repeat caesarean section. From the 617 eligible cases (failed TOLAC), 42 women were excluded due to missing data on pre-pregnancy or first-trimester weight gain. Using a systematic random sampling, every second case was started from the random number generated till we achieved the sample size of 200 cases. For the controls, every third case was included following systematic random sampling after excluding 31 women whose weight was not recorded due to antenatal booking only in the second trimester. 

Data collected

The information collected includes maternal age, education, occupation, parity, gestational age at the first antenatal visit, estimated fetal weight, and associated comorbidities. Previous delivery details such as indications for the last caesarean delivery, any previous successful VBAC or spontaneous vaginal deliveries, and any complications during labour and delivery. Information about labour and delivery in the current pregnancy, such as the onset of labour, mode of delivery, and any intra- or postpartum complications, was also collected.    

Sample size calculation

Based on the findings of Mei et al., who reported a 31% failure rate of TOLAC in women with excessive weight gain, and assuming a 45% failure rate in women with low or normal GWG, a sample size calculation was conducted [8]. With a power of 80%, an alpha error of 5%, and a 1:1 case-to-control ratio, it was determined that 188 participants per group would be required to detect significant differences. To account for potential dropouts or incomplete data, we enrolled 200 women in each group, resulting in a total sample size of 400 participants.   

Statistical analysis

Statistical analysis was performed using STATA 17.0 (StataCorp, College Station, TX). Continuous variables were summarised as either mean ± standard deviation or median (interquartile range), depending on the data distribution. Student’s t-test was used for normally distributed data to compare these variables between groups, and the Mann-Whitney U test for non-normally distributed variables. Depending on the data distribution, categorical variables were presented as frequencies and percentages, with group comparisons conducted using either the Chi-square test or Fisher’s exact test.  Logistic regression analysis assessed the relationship between excessive GWG and TOLAC outcomes. Univariate logistic regression, followed by multivariate logistic regression, was used to assess the potential confounders such as maternal age, parity, and the method of labour induction. The results of the multivariate analysis were expressed as adjusted odds ratios (aOR) with 95% confidence intervals (CIs), while controlling for potential confounding variables. Statistical significance was set at a *P*-value of <0.05. 

## Results

The mean maternal age of the cohort was 27.5 years, and the majority were primiparous (*n* = 346, 86.5%). The mean interpregnancy interval was 3.7 ± 2.1 years, with a history of VBAC noted in 46 (11.5%) patients. Most of them had a spontaneous onset of labour (*n *= 285, 71.2%). The mean gestational age at delivery was 38.5 ± 1.1 weeks. Birthweight was similar in both groups. Table 1 compares sociodemographic and clinical characteristics between those with successful and failed TOLAC. 

**Table 1:** Sociodemographic and clinical profile comparison between successful and failed TOLAC cases. Expressed as mean ± standard deviation, frequency (percentages), or # median. IQR, interquartile range; BMI, body mass index; LSCS, lower segment caesarean section; VBAC, vaginal birth after caesarean section; NICU, neonatal intensive care unit; TOLAC, trial of labour after caesarean

Characteristics	Total*	Successful TOLAC	Failed TOLAC*	𝜒2 or *t*-statistics	*P-*value
Age (years)	27.6 ± 4.1	27.1 ± 3.7	28.1 ± 4.4	2.407	0.014
Parity					
Primiparous	346 (86.5%)	163 (81.5%)	183 (91.5%)		0.003
Multiparous	54 (13.5%)	37 (18.5%)	17 (8.5%)	8.564	
Pre-pregnancy BMI					
Underweight (<18.5 kg/m^2^)	55 (13.7%)	34 (17.0%)	21 (10.5%)		0.003
Normal (18.5-24.9 kg/m^2^)	217 (54.3%)	117 (58.5%)	100 (50.0%)	11.036	
Overweight (>25 kg/m^2^)	128 (32.0%)	49 (24.5%)	79 (39.5%)		
Excessive weight gain (kg)					
No	176 (44.0)	91 (45.5)	85 (42.5)	0.365	0.546
Yes	224 (56.0)	109 (54.5)	115 (57.5)		
Comorbidities					
Hypertension in pregnancy	50 (12.5)	18 (36.0%)	24 (64.0%)	4.480	0.034
Gestational diabetes	32 (8.0)	9 (28.1%)	23 (71.9%)	6.658	0.010
Hypothyroid	35 (8.8)	13 (37.1%)	22 (62.9%)	2.530	0.111
Others	25 (6.3)	12 (48.0%)	13 (52.0%)	0.043	0.836
Prior obstetric details					
Maternal age at previous CS (years)	23.7 ± 3.8	23.2 ± 3.5	24.3 ± 3.9	2.866	0.004
Gestational age at previous LSCS (weeks)	38.6 ± 1.2	38.5 ± 1.3	39.0 ± 1.1	1.2890	0.198
Interpregnancy interval (years)^#^	3.8 ± 2.1	3.7 ± 2.2	3.8 ± 2.0	0.7151	0.475
Spontaneous vaginal delivery before LSCS	17 (4.3%)	9 (52.9%)	8 (47.1%)	0.610	0.804
Previous history of VBAC	46 (11.5%)	35 (76.0%)	11 (24.0%)	19.258	<0.001
Birth weight in previous pregnancy (kg)	2.9 ± 0.5	2.8 ± 0.5	2.9 ± 0.5	1.9529	0.051
Labour Details and Neonatal Outcomes					
Gestational age at delivery (weeks)	38.5 ± 1.2	38.4 ± 1.1	38.5 ± 1.3	0.2941	0.769
Onset of labour					
Spontaneous onset	285 (71.2%)	162 (56.9%)	123 (43.1%)	18.563	<0.001
Induced labor	115 (28.7%)	38 (33.1%)	77 (66.9%)		
Oxytocin augmentation	140 (35.0%)	46 (32.8%)	94 (67.2%)	25.319	0.001
Scar rupture	8 (2.0%)	0 (1.7%)	8 (4.0%)	8.1633	0.007
Blood transfusion	17 (4.3%)	7 (3.5%)	10 (5.0%)	0.553	0.457
Maternal ICU admission	16 (4.0%)	2 (1.0%)	14 (7.0%)	9.375	0.004
Birth weight (kg ± g)	2.933 ± 445.7	2.9277 ± 432.3	2.939.0 ± 459.6	0.251	0.801
NICU admission	22 (5.5%)	8 (36.4%)	14 (63.6%)	2.463	0.188
Hypoxic encephalopathy	4 (1.0%)	2 (50.0%)	2 (50.0%)	<0.001	>0.999
Respiratory distress	21 (5.3%)	8 (38.1%)	13 (61.9%)	1.256	0.262

The mean age of women with successful TOLAC was significantly lower than that of women in the failed group (27.1 years vs. 28.1 years; *P* = 0.014). The proportion of multiparous women was higher in those with a successful TOLAC (91.5% vs. 81.5%; *P* = 0.003). The distribution of women based on the pre-pregnancy BMI, hypertensive disorders of pregnancy, and gestational diabetes was significantly different between those who had success or failure after TOLAC (*P *< 0.05). More women with successful TOLAC had prior VBAC (76.0% vs. 24.0%; *P* < 0.001). Those who had induction labour (*P* < 0.001) and required oxytocin augmentation (*P* = 0.001) had more failures following TOLAC. Neonatal outcomes, including NICU admission (*P *= 0.188) and hypoxic encephalopathy (*P *> 0.999), did not differ significantly between successful and failed TOLAC groups. However, failed TOLAC was associated with higher rates of maternal ICU admission (*P *= 0.004) and uterine scar rupture (*P *= 0.007), emphasising the need for careful patient selection. 

Following multivariate analysis, after adjusting for other variables, excessive gestational weight gain was not associated with increased chances of failure following TOLAC compared to those with adequate or less weight gain during pregnancy (Table 2). 

**Table 2:** Logistic regression to assess the strength of association between various characteristics and TOLAC failure, after adjusting for the confounding factors presented in the table. VBAC, vaginal birth after caesarean; LSCS, lower segment caesarean section; BMI, body mass index; CI, confidence interval; TOLAC, trial of labour after caesarean

Parameter	Univariate, crude odds ratio (95% CI)	Multivariate, adjusted odds ratio (95% CI)*	*P*-value
Gestational weight gain			
Adequate or less	1.0	1.0	0.678
Excessive weight gain	1.17 (0.67-2.05)	1.09 (0.70-1.70)	
Pre-pregnancy BMI (kg/m^2^)	1.06 (1.02-1.12)	1.05 (1.00-1.10)	0.035
Maternal age (years)	1.06 (1.01-1.12)	1.08 (0.94-1.25)	0.269
Parity			
Primiparous	1.0	1.0	0.618
Multipara	1.09 (0.48-2.47)	1.34 (0.43-4.16)	
Maternal age, previous LSCS	1.07 (1.02-1.13)	-	-
Interpregnancy interval (years)	1.03 (0.94-1.14)	0.99 (0.85-1.19)	0.900
H/o vaginal delivery before LSCS			
No	1.0	1.0	0.649
Yes	0.88 (0.33-2.34)	0.73 (0.19-2.83)	
H/o VBAC			
No	1.0	1.0	0.004
Yes	0.21 (0.10-0.44)	0.18 (0.05-0.58)	
Comorbidities in the present pregnancy			
No	1.0	1.0	0654
Yes	1.96 (1.23-3.11)	1.13 (0.68-1.89)	
Gestational age delivery (weeks)	1.03 (0.86-1.21)	1.04 (0.86-1.24)	0.700
Induction of labour			
No	1.0	1.0	0.079
Yes	2.67 (1.70-4.20)	0.65 (0.94-2.88)	
Augmentation of labour			
No	1.0	1.0	0.004
Yes	2.96 (1.93-4.57)	2.16 (1.28-3.67)	

However, the increasing pre-pregnancy BMI (a 5 % increase with an increase in kg/m^2^), those who needed augmentation (aOR = 2.66) were independently associated with failure. In contrast, women with a prior history of VBAC had 82% lower odds of TOLAC failure compared with those without such a history.

## Discussion

Main findings  

Excessive maternal weight gain during pregnancy, according to the IOM guidelines, was not associated with increased chances of failure of trial of labour after caesarean section, adjusting for various factors in the study. The failure was increased with increasing pre-pregnancy BMI, and the odds doubled with augmentation of labour, whereas there was more than a two-thirds reduction in the odds of failure among those who had prior VBAC. Neonatal outcomes were similar among those with failed or successful TOLAC. 

Interpretation 

Studies assessing the impact of excessive GWG have reported contrasting results. Studies from New Zealand and the United States suggest that excessive GWG lowers the likelihood of a vaginal birth [9-11]. Some reported no significant association between them. Studies by both Mei et al. and Kominiarek and Peaceman reported that excessive GWG was not associated with higher rates of TOLAC failure, whereas increasing pre-pregnancy BMI increased the failure rates [8,12]. This finding is similar to that of the present cohort, in which excessive GWG was not significantly associated with failure, whereas increasing pre-pregnancy BMI was associated with a 5% increase in the odds of TOLAC failure for each 1 kg/m² increase. An increase in pre-pregnancy BMI is reported to reduce the success of TOLAC, probably following altered myometrial contractility, increased adipose-related inflammation, and a higher prevalence of comorbidities such as gestational diabetes and hypertensive disorders, all of which increase the chances of an intrapartum caesarean section [13-15]. One major challenge in suggesting GWG as a modifiable risk factor in many studies is that it could be calculated at admission for delivery. However, the pre-pregnancy BMI could be modified if pregnancy is planned after reducing the weight, thus increasing the probability of success of TOLAC. 

Similar to earlier studies, those with a prior successful VBAC reduced the odds of failure by 82% [16,17]. Furthermore, labour interventions such as oxytocin augmentation were associated with increased risk of TOLAC failure (aOR = 2.66), reinforcing concerns around uterine contractile dysfunction in women with higher BMI or prolonged labour and reduced response to oxytocin [18]. The absence of significant differences in key neonatal outcomes (NICU admission, hypoxic encephalopathy) between groups suggests that maternal risks, particularly uterine rupture and ICU admission, should be prioritised in decision-making for TOLAC eligibility. 

One of the strengths of this study lies in its prospective design, which reduces recall bias and ensures accurate, consistent data collection, thereby enhancing the overall robustness of the findings. Additionally, the multivariate analysis allowed for adjustment of key confounders such as maternal age, parity, and previous VBAC, contributing to the internal validity of the results. However, the study also presents certain limitations. Being conducted at a single centre, the findings may not be generalisable to broader populations, particularly those in different geographic or socioeconomic contexts. The sample size limited the ability to stratify the patients based on the pre-pregnancy BMI, potentially limiting the assessment of interaction between the pre-pregnancy BMI strata and the excessive weight gain on the outcome of TOLAC. Since we used a pragmatic real-world approach, where the patients were referred to the tertiary centre at various gestations, the variability in weight measurement scales across different centres introduces potential measurement bias. Future research should address these limitations by incorporating larger populations stratified by the pre-pregnancy BMI and utilising standardised, calibrated measurement protocols to improve the validity and generalisability of findings. 

## Conclusions

In this cohort of women with a previous CS, there was no significant association between excessive GWG and failure after the trial of labour. However, higher pre-pregnancy BMI and labour augmentation were linked to increased failure risk, while prior successful VBAC increases the chances of success. These results highlight the importance of preconception weight management and careful intrapartum planning.
